# 

**Published:** 1924-02

**Authors:** 


### ORGANISING A HEALTH WEEK.

### A PRIZE OF £100.

"HEALTH Weeks" are a comparatively new departure. Here and there a great town has organised such a week, and there has recently been a very successful one in the Borough of St. Pancras, originated by the Acting Medical Officer of Health, Dr. Stella Churchill. Efforts of this kind are, however, still novel and distinctly occasional. To most people, indeed, the very name is unfamiliar. Yet all manner of "Weeks" and "Days" are constantly being organised for purposes which, however admirable, are mostly of small moment compared with the primary social subject of the health of the people. The tate devotes a more minute attention than ever before to the physical well-being of the nation, but we are a sturdy and independent race, and we love to do things for ourselves and not to be beholden to Governments when by individual initiative we can help ourselves. The laws of health must needs be emphasised by the laws of the realm, but there is much that can be done outside Governments to make the sanitary code laid down by Nature better known and more carefully followed. We are only beginning to recognise the imperious character of this code, the observance of which would, in comparatively a few years, add enormously to the health, happiness and well-being of the people.

There is no better, no readier, no more popular means of impressing upon the public mind the duty, social and religious, of doing everything that in us lies—and every intelligent person can do much—not merely to reduce disease, but to prevent it. The medicine of the future will be mainly preventive medicine, and it is to prevention that "Health Weeks" primarily look. To teach the people how to preserve health, how to safeguard infant life, how to push death from disease farther and farther away is a noble object. And that, put into a nutshell, is the object of a "Health Week." Who, then, shall deny that there ought in every community in the country to be such a Week, always full of varied and fascinating interest, and always increasing in scope and attraction ?

It is the conviction of THE HOSPITAL AND HEALTH REVIEW that "Health Weeks" are destined to increase rapidly in number and to exercise a wide and powerful influence over the mind of the people. But they require to be carefully thought out, their details to be considered in the light of experience, and the incidents which compose them arranged with an eye to picturesque appeal. The newspaper, the pictorial poster the lecture, the film, the pageant, wireless, even music and singing can, and ought to be, pressed into the service. A Week of this kind should be a joyous Carnival, an event to be looked forward to with animated expectancy, and looked back upon with all the satisfaction of happy realisation. Instruction should be so imparted that a town or a district should long for the opportunity of acquiring knowledge of the laws of health and how to observe them. A dull or monotonous "Health Week" is certain to be a failure.

In the hope and in the confident expectation of popularising and greatly increasing the number of "Health Weeks" THE HOSPITAL AND HEALTH REVIEW is offering a prize of One Hundred Pounds for the best and most practical answer to the following three simple questions :—

1. How would you start a "Health Week" in your town or district ?
2. What would you make the principal features (naming not more than three) and why ?
3. How would you insure the success of your "Health Week" ?

We do not prescribe any length for these answers. They may be short or long, as the competitor pleases, but it is obvious that the most effective answers are likely to be crisp and compendious. A competitor may send in as many replies as he pleases, but each one must be accompanied by the coupon which will be found at the foot of page 64 of this issue. The competition will remain open until March 25, and we wish it to be clearly understood that it is open to all the world, men and women, doctors and laymen, health visitors and nurses. Everyone who has ideas on the subject is invited to give us the benefit of them- Each answer must bear the name and address of the competitor legibly inscribed. The best answer will be published, together with the name, address and portrait of the winner in our May number, which will be on sale on April 25. We reserve the right to publish any other answer which may be deemed to be sufficiently excellent. The decision of the Editor of THE HOSPITAL AND HEALTH REVIEW is to be accepted as final and conclusive.

### ANSWERS TO CORRESPONDENTS.

S.S.—We know of no book on Cottage hospitals published since the war.

Reader.—Food can only be kept really hot in such circumstances by the use of a handy food trolley. Try Messrs. Benham and Sons, of 64-66 Wigmore Street, whose "Foxborough" Insulated Food Trolley is well thought of.

M. D.—Whooping-cough was a familiar disease in the sixteenth century, as we know from a reference to it by Baillou of Paris in 1578.

B. W.—We should recommend you to communicate with a firm which specialises in this direction, and to write to Messrs. Dunn, Bennett and Co., of the Royal Victoria Pottery, Burslem, who are extensive manufacturers of crockery for hospitals and institutions generally.

Enquirer.—No; in-patients without a legal settlement in the County of London cannot be accepted at the Maudsley Hospital for less than £5 per week.

John.—Yes, Paripan Enamel is largely used for the purpose as it will stand any amount of washing. All particulars respecting it can be obtained from Paripan, Ltd., Sherwood House, Piccadilly Circus, W.

J. H.—Write to the Secretary of the Health Week Committee, at the Royal Sanitary Institute, 90 Buckingham Palace Road, S.W. 1.

Sutures.—We believe so. C. F. Thackeray, of 119, High Holborn, W.C. 1, and Great George Street, Leeds, is the wholesale distributor of D. and G. Sutures.

R. L.—The aiticle, "Should the Doctor Tell?" appeared in The Hospital of December 22, 1917.

### RECENT APPOINTMENTS.

### Medical Officers, Chaplains, &c.

Acland, T. D., M.R.C.S., L.R.C.P.; Honorary Consulting Physician, Ministry of Pensions.

Beatty, C. C., M.C., M.R.C.P., Physician (wth charge of Out-Patients), Royal Northern Hospital.

Bisset, Mary; M.O. for the County Council Schools, Brighton.

Dunscombe, N. D., M.D., Ch.B., D.P.H.; Casualty Officer, East London Hospital for Children and Dispensary for Women, Shadwell.

Forward, F. E., M.R.C.S., L.R.C.P., M.O. Maidstone Prison; M.O. Parkhurst Prison.

Grant, M.A., M.B., Ch.B.; Assistant School M.O. Stockport District of Cheshire.

Graham, George, M.D., F.R.C.P., Physician (with charge of In-Patients), Royal Northern Hospital.

Göthe, Werner, M.R.C.S., L.R.C.P.; M.O. in charge of Physics and Electro-Therapeutic Dept., Middlesex Hospital.

Hembrow, C. H., M.B., House Surgeon, Royal Northern Hospital.

Kinloch, John Parlane, M.D., D.P.H., Deputy M.O.H.' Aberdeen; M.O.H., Aberdeen.

McCoy, H. A., M.B., Ch.M., Assistant Radiologist, Royal Northern Hospital.

Maconie, A. C., M.B., House Surgeon, Royal Northern Hospital.

Mason, G. A., M.B., Casualty Officer, Royal Northern Hospital.

Paton Richard, R.E., M.B., D.P.H., Assistant M.O., Glasgow; M.O.H. and School M.O., St. Albans Rural District.

Rose, W. G., F.R.C.S., Resident Medical Office, Royal Northern Hospital.

Rosuwarne, D. D., M.R.C.S., L.R.C.P., Assistant M.O., City of London and East London Dispensary.

Stefle, R. U., M.B., B.S.; Post Office M.O. for Camden Town Division.

White, E. B., M.R.C.S., L.R.C.P., Deputy Medical Superintendent Dorset County Mental Hospital; Medical Superintendent Bristol Mental Hospital.

### Public Health and Hospital Appointments.

Allbutt, Mary Gladys, Matron, Fulham Infirmary.

Andrews, A. C.; Welfare Centre Sister, Shoreditch.

Aspinall, Elizabeth; Matron, Hayes School for Jewish Boys.

Beancarlari, Emily, Sister Devon and East Cornwall Hospital; Matron, Infectious Diseases Hospital, Elgin.

Betteridge, Edith; Child Welfare and School Nurse, Tipton.

Ellis, Edith Gertrude, Health Visitor, Dewsbury.

Foster, G. R., Assistant County Council Divisional Surveyor, Okehampton; Sanitary Inspector, Crediton.

Green, E. M., Lady Almoner, Royal Northern Hospital; Lady Almoner, King's College Hospital.

Hirst, Margaret E., Matron Houldsworth Homœopathic Hospital, Glasgow; Matron Dover Royal Victoria Hospital.

Howes, E. M., St. Thomas's Hospital; Lady Almoner, Royal Northern Hospital.

Jackson, D., Assistant Health Visitor, Belgrave Hospital Infant Welfare Centre.

Maguire, Grace E., C.M.B.; Health Visitor, Canterbury.

Murphy, B., Health Visitor and School Nurse, Winchester.

Paris, F. E.; School Nurse, Hornsey.

Penney, Miss, A.I.H.A., Assistant-Almoner at the East London Hospital for Children, Shadwell, E.

Phillips, Russell, M.B.; M.O.H., Madron, Cornwall.

Pollok, Joan M.; Maternity and Child Welfare Nurse, Greenock.

Price, Alice; Health Visitor, Burnley.

Queensworth, Elsie, Health Visitor, Liverpool; Matron, Carnegie Infant Welfare Centre, Liverpool.

Rolls, H. A.; Surveyor and Sanitary Inspector, Linslade.

Scrimgeour, Miss, A.I.H.A.; Almoner at the Fleming Hospital, Newcastle-on-Tyne.

Simpson, Grace, M.A.; Health Visitor, Lewisham.

Soames, R. M., M.R.C.S., L.R.C.P., M.O.H., Warblington.

Tupper, Miss, A.I.H.A.; Almoner of the Infants' Hospital, Vincent Square, S.W. 1.

Wardle, E. A., C.M.B.; District Nurse, Midwife, Health Visitor and School Nurse, Wicken and Deanshanger Nursing Association.

Watt, J. P., Superintendent Fife County Nursing Association; Organiser for Scotland, Queen Victoria Jubilee Nurses' Institute.

Willey, B. M., Matron, Bexley Health and District Cottage Hospital.

Williams, Emily F.; Assistant School and Dental Nurse, Beckenham.

### COTTAGES FOR THE TUBERCULOUS.

Professor S. Lyle Cummins, of the University of South Wales, speaking on "Tuberculosis: How and When is it an Infective Disease?" at the Royal Institute of Public Health, said there were some people who contended that persons could be thrown into very close association with infected people without catching the disease and others who advocated complete segregation for tuberculosis cases. The truth, however, was between the two. Infection should not be confused with disease. He gave figures to show the high percentage of cases of tuberculosis which came from infected families. People were relatively safe if they were already infected, but if exposed to infection on a large scale their resistance broke down. Complete segregation would not do, as it would lead to concealment of disease. He advocated building special cottages in the country for tuberculous patients and their families if so desired. It would pay the country to do this, as tuberculosis was a great menace to the health of the community.

"THE HOSPITAL AND HEALTH REVIEW"

### Managerial Notices.

*All letters on Business matters must be addressed to the Manager, "The Hospital and Health Review," 28 and 29 Southampton Street, London, W.C. 2.*

Rates for Subscription (payable in advance).

| | | |
|---|---|---|
| Three Months (including Postage) | .. .. | 1s. 9d. |
| Six Months do. | .. .. | 3s. 6d. |
| Twelve Months do. | .. .. | 7s. 0d. |

**It is especially requested that remittances be made by Cheque or Postal Order and not Stamps.**

Subscriptions, which may commence at any time, are payable in advance. Cheques and Post-Office Orders should be crossed Westminster Bank, Covent Garden Branch, and made payable to the Manager of The Hospital and Health Review.

***Advertisements.*** Scale of charges and particulars of special positions may be obtained on application to the Manager.

*SUBSCRIPTION FORM.*

*Please send me* The Hospital and Health Review *for*............. *months, commencing with your issue of* ............. *for which please find enclosed* .......

*Name* ........................................

*Address* ........................................

